# Hepatitis B reactivation risk and physician awareness in rheumatological patients receiving anti-tumor necrosis factor-α treatment

**DOI:** 10.1590/1806-9282.20240091

**Published:** 2024-07-19

**Authors:** Osman Cure, Bayram Kizilkaya, Serdar Durak, Kadir Ilkkilic

**Affiliations:** 1 Recep Tayyip Erdogan University, School of Medicine, Department of Rheumatology – Rize, Turkey.; 2 Recep Tayyip Erdogan University Training and Research Hospital, Internal Medicine – Rize, Turkey.; 3 Bitlis State Hospital, Department of Gastroenterology – Bitlis, Turkey.; 4 Recep Tayyip Erdogan University, School of Medicine, Department of Hematology – Rize, Turkey.

**Keywords:** Rheumatic diseases, Tumor necrosis factor alpha inhibitor, Hepatitis B virus

## Abstract

**OBJECTIVE:**

We aimed to evaluate the risk of hepatitis B virus reactivation in rheumatic patients using anti-tumor necrosis factor-alpha drugs and the awareness of physicians about hepatitis B virus reactivation.

**METHODS:**

Demographic characteristics, pre- and post-treatment hepatitis markers, and laboratory parameters of patients receiving anti-tumor necrosis factor-alpha therapy in our rheumatology clinic were retrospectively examined.

**RESULTS:**

A total of 448 patients, 240 (53.6%) female and 208 (46.4%) male, were evaluated. Their mean age was 48.02±14.64 years. While HBsAg was examined in 443 (98.9%) patients before treatment, 7 (1.6%) patients were found to be HBsAg positive. While anti-HBc IgG was examined in 405 (90.4%) patients, it was positive in 69 (17%) patients. HBs Ag (total 446–99.6%) test was performed in three patients who were not tested for HBsAg before the treatment, and anti-HBc total (431–96.2% total) test was performed in 26 patients who were not tested for anti-HBc total. All HBsAg positive patients and 17 (24.6%) of those with previous hepatitis B received antiviral treatment. While the median follow-up period of the patients was 24 (6–60) months, no patient developed hepatitis B virus reactivation.

**CONCLUSION:**

The screening rates and awareness of physicians providing anti-tumor necrosis factor-alpha therapy for hepatitis B virus infection were found to be higher compared to similar studies. Hepatitis B virus reactivation did not develop in any patient. Since the risk of hepatitis B virus reactivation is low, especially in patients with previous hepatitis B, it would be more appropriate to follow up the patients without giving antiviral prophylaxis.

## INTRODUCTION

Anti-tumor necrosis factor-alpha (TNF-α) and disease-modifying antirheumatic drugs (DMARDs) are used in the treatment of various rheumatic diseases, especially rheumatoid arthritis (RA)^1^. ’Traditional DMARDs used in rheumatic treatment include methotrexate (MTX), hydroxychloroquine, sulfasalazine and leflunomide. DMARDs with potential hepatotoxic and immunosuppressive effects, such as MTX, can cause activation of the hepatitis B virus (HBV). In addition to these drugs, combining steroids with anti-TNF-α and other biologicals poses a risk for HBV activation^2^. TNF-α plays an important role in host defense. Patients treated with anti-TNF-α agents have increased susceptibility to infections. TNF-α is a cytokine that can suppress HBV replication and has an important role in the elimination of HBV by stimulating HBV-specific cytotoxic T-cell responses^3^. Prophylactic antiviral therapy has proven to be of significant benefit in preventing HBV reactivation in HBsAg positive patients treated with anti-TNF-α agents or DMARDs^4^. Therefore, it is recommended to initiate antiviral prophylaxis or treatment for chronic HBV infection in rheumatic patients receiving anti-TNF-α therapy or DMARDs^2^.

In this study, we aimed to evaluate the HBVr risk and physician awareness of HBVr in patients using anti-TNF-α due to inflammatory rheumatological disease.

## METHODS

### Study population

The data of patients who received anti-TNF-α treatment at the rheumatology outpatient clinic of Recep Tayyip Erdoğan University Training and Research Hospital between June 2018 and June 2023 were retrospectively examined.

### Data collection

Using our hospital's electronic record system, the patients' diagnoses, demographic characteristics, anti-TNF-α starting dates and treatment durations, HBV serology before and after anti-TNF-α treatment, and clinical and laboratory results of the patients were evaluated.

### Exclusion criteria

Patients who received anti-TNF-α treatment for less than 2 months, patients under 18 years of age, cancer patients, patients with insufficient viral marker data, patients without clinical follow-up and laboratory results, and those who were found to be positive for hepatitis C virus RNA were excluded from the study.

### Definitions

HBsAg and/or anti-HBc tests were performed within 6 months before the start of treatment with anti-TNF-α drugs, HBV screening, and patients with HBsAg positivity detected for more than 6 months were defined as chronic hepatitis B patients. HBsAg negative/anti-HBc positive patients are defined as having recovered from HBV infection^5,6^. While monitoring patients, HBV reactivation was defined as a 10-fold increase in viral load from baseline negative to HBV DNA positivity and/or a change from baseline negative to HBsAg positivity^5-7^. Hepatitis was defined as an increase in the serum alanine aminotransferase (ALT) level of at least three times the upper limit of normal (45 U/L for serum ALT)^6^. HBV-related hepatitis was defined as clinical and biochemical evidence of hepatitis with an increase in HBV DNA^5^.

### Serological and virological evaluation for hepatitis B virus infection

Hepatitis B virus serological markers, including HBsAg, anti-HBs, and anti-HBc levels, were evaluated by electrochemiluminescence immunoassay method using the Roche Cobas e6001 device (Roche Diagnostics, Mannheim, Germany). Serum HBV DNA levels were measured by Rotor-Gene Q (QIAGEN, Hilden, Germany) real-time polymerase chain reaction method (lower detection limit was 12 IU/mL). Routine biochemical parameters were tested using the Roche Hitachi Cobas 8000 Modular Analyzer system (Roch Diagnostics, Germany).

### Statistical analysis

The SPSS Windows version 22 program was used in statistical tests. Continuous variables were evaluated for normal distribution with histogram, Q–Q graph, and Shapiro-Wilk or Kolmogorov-Smirnov test depending on the number of variables. Among continuous variables, those with normal distribution were presented as mean±standard deviation throughout the entire study, and independent variables t-test was used to compare the two groups. Other continuous variables were presented with median (minimum–maximum) values, and the non-parametric Mann-Whitney U test was used to compare the groups. Categorical variables were presented as frequency and percentage, and Pearson chi-square test or Fisher's exact probability test was used to compare the groups. Tests with a p-value of 0.05 or less within the 95% confidence interval were considered statistically significant.

## RESULTS

A total of 448 patients were included in the study, of whom 240 (53.6%) patients were female and 208 (46.4%) were male. The average age of the patients was 48.02±14.64 years, and the average age of women was significantly higher than that of men (p<0.001) (Table 1).

**Table 1:** Demographic characteristics of the patients.

Variable		P
Male/female, n (%)	208 (46.4)/240 (53.6)	
Age, mean±SD	48.02±14.64	**<0.001** a
Male	43.78±14.56	
Female	51.70±13.71	

a T-test, SD: standard deviation.

Statistically significant value is denoted in bold.

Patients received anti-TNF-α treatment due to 226 (50.4%) ankylosing spondylitis, 160 (35.7%) RA, 54 (12.1%) psoriatic arthritis, and 8 (1.8%) Behçet's disease. Notably, 150 (33.5%) patients received etanercept, 137 (30.6%) received golimumab, 112 (25%) received adalimumab, 33 (7.4%) received infliximab, and 16 (3.6%) received certolizumab treatment. The most common comorbid conditions found in the patients were hypertension in 118 (26.3%), diabetes in 37 (8.3%), coronary artery disease in 26 (5.8%), hyperlipidemia in 12 (2.7%), and chronic obstructive pulmonary disease in 8 (1.8%).

HBsAg was examined in 443 (98.9%) patients before treatment. While anti-HBc IgG was examined in 405 (90.4%) patients, it was not examined in 43 (9.6%) patients. Anti-HBc IgG test was positive in 69 (17%) patients. HBsAg (total 446–99.6%) test was performed in three patients who were not tested for HBsAg before the treatment, and an anti-HBc total (431–96.2% total) test was performed in 26 patients who were not tested for anti-HBc total. While the total number of HBsAg positive patients was 7 (1.6%), the number of HBsAg negative/anti-HBc total positive patients was 69 (16%).

A total of 24 patients received antiviral treatment. In addition, 11 patients received entecavir, 12 received tenofovir disoproxil fumarate (TDF), and 1 received lamivudine treatment. Antiviral treatment was started prophylactically in all HBsAg positive patients, 1 with entecavir and 6 with TDF. Antiviral treatment was started prophylactically in 17 (24.6%) patients, including entecavir in 10 patients, TDF in 6 patients, and lamivudine in 1 patient with HBsAg negative and anti-HBc total positive.

### HBsAg negative/anti-HBc total positive patients

The average age of 69 patients with HBsAg negative/anti-HBc total positive was 54.33±13.07 years and 45 of them (65.2%) were women. The average age of women was higher than men (p=0.016). The median follow-up period was 24 (6–60) months. Of the patients receiving anti-TNF-α therapy, RA was noted in 30 (43.5%), ankylosing spondylitis in 29 (42%), psoriatic arthritis in 7 (10.1%), and Behçet's disease in 3 (4.3%). The treatment received by the patients was 29 (42) golimumab, 22 (31.9%) etanercept, 13 (18.8%) adalimumab, 4 (5.8%) infliximab, and 1 (1.4%) certolizumab (Table 2). While HBV DNA test was performed in 22 (31.9%) patients before treatment, all of them were found to have negative HBV DNA levels.

**Table 2:** Demographic characteristics of HBsAg negative/anti-HBc total positive patients.

Variable			P
Male/female, n (%)		24 (45.8)/45 (65.2)	
Age, mean±SD		54.33±13.07	**<0.016** a
Male		49.21±14.79	
Female		57.07±11.31	
Diagnosis, n (%)			
	Rheumatoid arthritis	30 (43.5)	
	Ankylosing spondylitis	29 (42)	
	Psoriatic arthritis	7 (10.1)	
	Behcet's disease	3 (4.3)	
Treatment, n (%)			
	Golimumab	29 (42)	
	Etanercept	22 (31.9)	
	Adalimumab	13 (18.8)	
	Infliximab	4 (5.8)	
	Sertozulimab	1 (1.4)	

a T-test, SD: standard deviation.

Statistically significant value is denoted in bold.

## DISCUSSION

Hepatitis B infection is one of the most common infections worldwide. Chronic hepatitis B infection is an important cause of morbidity and mortality in society, leading to the development of hepatocellular cancer and cirrhosis. HBVr occurs with the reactivation of the patients' immune response against HBV. In cases of immunosuppression from any cause, immune-mediated control of HBV replication is impaired and reactivation may subsequently occur. Anti-TNF-α inhibitors, steroids, and other immunosuppressive drugs used in rheumatological diseases may cause the functions of T and B lymphocytes to deteriorate, thus suppressing the immune response to HBV^8,9^.

In a study conducted by Lan et al., it was reported that 40% of chronic hepatitis B patients developed HBVr due to the use of anti-TNF-α, and 5% of them had a mortality risk^10^. In another study, the HBVr rate was found to be between 27 and 39% in HBsAg positive patients receiving anti-TNF-α. In the study conducted by Pérez Alvarez et al., HBVr was reported in 35 (39%) out of 87 HBsAg positive patients receiving anti-TNF-α and in 9 (5%) out of 168 HBsAg negative/anti-HBc positive patients. One of the HBsAg negative/anti-HBc positive patients died due to fulminant liver failure^11,12^. In a study, it was reported that in patients receiving anti-TNF-α therapy with an indication of autoimmune disease, 9 out of 23 patients (39%) who were HBsAg positive at the beginning of treatment developed HBVr, but none of the 178 HBsAg negative/anti-HBc positive patients developed HBVr^7^. In our study, no patient developed HBVr, regardless of hepatitis serology and antiviral prophylaxis.

The Asian Pacific Association for the Study of the Liver and the American Association for the Study of Liver Diseases guidelines recommend prophylactic antiviral treatment during treatment and for up to 12 months after discontinuation of treatment in all HBsAg positive patients receiving immunosuppressive therapy, regardless of HBV DNA level. The European Association for the Study of Liver Diseases Study of the Liver recommends preemptive treatment during treatment and for up to 12 months after discontinuation of treatment in all HBsAg positive patients who are candidates for immunosuppression or chemotherapy, regardless of HBV DNA level. Regarding HBsAg negative/anti-HBc positive patients, all guidelines recommend that if the HBV DNA level is detectable, the patients should be treated as if they were HBsAg positive^13-15^. In our study, prophylactic antiviral treatment was started in all 7 HBsAg positive patients and in 17 (24.6%) HBsAg negative/anti-HBc total positive patients, even though 10 of them had negative HBV DNA levels and 7 of them had not had HBV DNA levels checked.

Most reported cases of HBVr in HBsAg negative/anti-HBc positive patients occurred in patients receiving concomitant use of anti-TNF-α therapy and other immunosuppressive drugs^16,17^. In our study, although 32 (46.3%) of the negative/anti-HBc positive patients were receiving steroid treatment before treatment, no patient developed reactivation.

Guidelines recommend that patients who will be given anti-TNF-α therapy should be screened for hepatitis B. HBsAg, anti-HBc, and anti-HBs should be checked in these patients. If HBsAg and/or anti-HBc are positive, HBV DNA should be examined. HBV seronegative patients should be vaccinated. Those diagnosed with chronic hepatitis B should be evaluated for antiviral treatment for hepatitis^17-19^. In our study, before starting anti-TNF-α treatment in rheumatological patients, screening was performed in a very high proportion of patients compared to the literature, although not all patients were screened according to guideline recommendations. In addition, patients in whom HBsAg/anti-HBc total was not considered during the anti-TNF-α treatment were examined for screening during follow-up. In our study, 98.9% patients were suggested for HBsAg test and 90.4% patients were suggested for anti-HBc total test.

### Limitations

As a single-centered and retrospective study, our failure to access all data is the limitation of our study. Failure to follow up the majority of patients in terms of HBV DNA level limits the study.

## CONCLUSION

Anti-TNF-α treatment of physicians in terms of HBV infection rates and awareness of the ratio were found to be higher than similar studies. HBVr has not developed in any patient who has been passed by hepatitis B and did not receive antiviral treatment. Since the risk of HBVr is low in such patients, it will be appropriate to follow up patients with gastroenterological or infectious diseases without giving antiviral professional physicians and to increase the awareness of physicians who provide immunosuppressive treatment for HBVr.

## ETHICS COMMITTEE APPROVAL

The study was conducted in accordance with the principles of the Declaration of Helsinki and was approved by the Recep Tayyip Erdoğan University Local Ethics Committee (No. 2023/209).

## References

[1] Li J, Zhang Z, Wu X, Zhou J, Meng D, Zhu P (2021). Risk of adverse events after anti-TNF treatment for inflammatory rheumatological disease. A meta-analysis. Front Pharmacol.

[2] Fragoulis GE, Nikiphorou E, Dey M, Zhao SS, Courvoisier DS, Arnaud L (2023). 2022 EULAR recommendations for screening and prophylaxis of chronic and opportunistic infections in adults with autoimmune inflammatory rheumatic diseases. Ann Rheum Dis.

[3] Vassilopoulos D, Calabrese LH (2013). Viral hepatitis: review of arthritic complications and therapy for arthritis in the presence of active HBV/HCV. Curr Rheumatol Rep.

[4] Chiu YM, Chen DY (2020). Infection risk in patients undergoing treatment for inflammatory arthritis: non-biologics versus biologics. Expert Rev Clin Immunol.

[5] Myint A, Tong MJ, Beaven SW (2020). Reactivation of hepatitis B virus: a review of clinical guidelines. Clin Liver Dis (Hoboken).

[6] Mori S, Fujiyama S (2015). Hepatitis B virus reactivation associated with antirheumatic therapy: risk and prophylaxis recommendations. World J Gastroenterol.

[7] Pauly MP, Tucker LY, Szpakowski JL, Ready JB, Baer D, Hwang J (2018). Incidence of hepatitis B virus reactivation and hepatotoxicity in patients receiving long-term treatment with tumor necrosis factor antagonists. Clin Gastroenterol Hepatol.

[8] Shi Y, Zheng M (2020). Hepatitis B virus persistence and reactivation. BMJ.

[9] Loomba R, Liang TJ (2017). Hepatitis B Reactivation associated with immune suppressive and biological modifier therapies: current concepts, management strategies, and future directions. Gastroenterology.

[10] Lan JL, Chen YM, Hsieh TY, Chen YH, Hsieh CW, Chen DY (2011). Kinetics of viral loads and risk of hepatitis B virus reactivation in hepatitis B core antibody-positive rheumatoid arthritis patients undergoing anti-tumour necrosis factor alpha therapy. Ann Rheum Dis.

[11] Ostuni P, Botsios C, Punzi L, Sfriso P, Todesco S (2003). Hepatitis B reactivation in a chronic hepatitis B surface antigen carrier with rheumatoid arthritis treated with infliximab and low dose methotrexate. Ann Rheum Dis.

[12] Pérez-Alvarez R, Díaz-Lagares C, García-Hernández F, Lopez-Roses L, Brito-Zerón P, Pérez-de-Lis M (2011). Hepatitis B virus (HBV) reactivation in patients receiving tumor necrosis factor (TNF)-targeted therapy: analysis of 257 cases. Medicine (Baltimore).

[13] Terrault NA, Lok ASF, McMahon BJ, Chang KM, Hwang JP, Jonas MM (2018). Update on prevention, diagnosis, and treatment of chronic hepatitis B: AASLD 2018 hepatitis B guidance. Hepatology.

[14] Sarin SK, Kumar M, Lau GK, Abbas Z, Chan HL, Chen CJ (2016). Asian-Pacific clinical practice guidelines on the management of hepatitis B: a 2015 update. Hepatol Int.

[15] European Association For The Study Of The Liver (2012). EASL clinical practice guidelines: management of chronic hepatitis B virus infection. J Hepatol.

[16] Watanabe T, Fukae J, Fukaya S, Sawamukai N, Isobe M, Matsuhashi M (2019). Incidence and risk factors for reactivation from resolved hepatitis B virus in rheumatoid arthritis patients treated with biological disease-modifying antirheumatic drugs. Int J Rheum Dis.

[17] European Association for the Study of the Liver (2017). EASL 2017 clinical practice guidelines on the management of hepatitis B virus infection. J Hepatol.

[18] Gonzalez SA, Perrillo RP (2016). Hepatitis B virus reactivation in the setting of cancer chemotherapy and other immunosuppressive drug therapy. Clin Infect Dis.

[19] Terrault NA, Lok ASF, McMahon BJ, Chang KM, Hwang JP, Jonas MM (2018). Update on prevention, diagnosis, and treatment of chronic hepatitis B: AASLD 2018 hepatitis B guidance. Hepatology.

